# Supplementary material for: MetaRibo-Seq measures translation in microbiomes
Source: Nat Commun. 2020 Jun 29;11:3268. doi: 10.1038/s41467-020-17081-z (PMC7324362; doi:10.1038/s41467-020-17081-z)
Supplement: Supplementary file 10 — Supplementary Data 7 [file 41467_2020_17081_MOESM10_ESM.zip › File2/Confidence_High_Taxonomy/199696_out.krona.html]

Javascript must be enabled to view this page.

members
magnitude
magnitudeUnassigned
count
unassigned
taxon
rank

199696\_out

56

56
superkingdom
2

56
phylum
1239

class
5
186801

order
5
186802

family
2
186803

572511
genus
1

1

SRS063040\_contig\_number\_45945
species
2292967

1898203

SRS075773\_contig\_number\_9251
species
1

31979
family
1

1485
genus
1

species

SRS047044\_contig\_number\_3800
1
1262813

1
family
541000


SRS1041136\_contig\_number\_4657
species
1
39492

1898207
1
species

SRS015782\_contig\_number\_43234

91061
class
3

order
3
186826

3
family
186828

2747
genus
3


SRS077552\_contig\_number\_28543SRS1041095\_contig\_number\_5706SRS143991\_contig\_number\_19615
species
3
1637504

909932
48
class

1843488
48
order

family
48
909930

33024
genus
48

3
species

SRS016095\_contig\_number\_contig-100\_208.160039SRS098717\_contig\_number\_contig-100\_344.128316SRS971275\_contig\_number\_43000
1262914


SRS011084\_contig\_number\_22660SRS012273\_contig\_number\_contig-100\_476.273589SRS012902\_contig\_number\_322SRS012969\_contig\_number\_18966SRS014412\_contig\_number\_18594SRS014979\_contig\_number\_21223SRS021484\_contig\_number\_11449SRS022071\_contig\_number\_34861SRS022137\_contig\_number\_18792SRS022713\_contig\_number\_5399SRS023526\_contig\_number\_20845SRS023829\_contig\_number\_344SRS049773\_contig\_number\_17725SRS050941\_contig\_number\_21351SRS053214\_contig\_number\_21927SRS053335\_contig\_number\_2904SRS056519\_contig\_number\_12939SRS062701\_contig\_number\_5431SRS065176\_contig\_number\_10156SRS074670\_contig\_number\_contig-100\_305.61959SRS077127\_contig\_number\_14989SRS077730\_contig\_number\_16504SRS077849\_contig\_number\_12662SRS097889\_contig\_number\_36333SRS098644\_contig\_number\_42933SRS1041091\_contig\_number\_contig-100\_30.119847SRS1041134\_contig\_number\_1868SRS1041137\_contig\_number\_20472SRS105153\_contig\_number\_14149SRS1055067\_contig\_number\_15826SRS1055099\_contig\_number\_5607SRS142503\_contig\_number\_29451SRS143598\_contig\_number\_4966SRS143722\_contig\_number\_8982SRS143780\_contig\_number\_31251SRS144362\_contig\_number\_25566SRS146813\_contig\_number\_23294SRS148196\_contig\_number\_38193SRS148319\_contig\_number\_11909SRS148511\_contig\_number\_18056SRS148721\_contig\_number\_19404SRS148874\_contig\_number\_6763SRS893231\_contig\_number\_394SRS893270\_contig\_number\_23918SRS893295\_contig\_number\_12149
species
45
33025
